# Automated, real-time material detection during ultrashort pulsed laser machining using laser-induced breakdown spectroscopy, for process tuning, end-pointing, and segmentation

**DOI:** 10.1371/journal.pone.0290761

**Published:** 2024-01-12

**Authors:** Hongbin Choi, Adrian Phoulady, Pouria Hoveida, Nicholas May, Sina Shahbazmohamadi, Pouya Tavousi

**Affiliations:** University of Connecticut, Storrs, Connecticut, United States of America; York St John University, UNITED KINGDOM

## Abstract

The rapid, high-resolution material processing offered by ultrashort pulsed lasers enables a wide range of micro and nanomachining applications in a variety of disciplines. Complex laser processing jobs conducted on composite samples, require an awareness of the material type that is interacting with laser both for adjustment of the lasering process and for endpointing. This calls for real-time detection of the materials. Several methods such as X-ray diffraction (XRD), X-ray photoelectron spectroscopy (XPS), and energy dispersive X-Ray spectroscopy (EDS) can be used for material characterization. However, these methods often need interruption of the machining process to transfer the sample to another instrument for inspection. Such interruption significantly increases the required time and effort for the machining task, acting as a prohibitive factor for many laser machining applications. Laser induced breakdown spectroscopy (LIBS) is a powerful technique that can be used for material characterization, by analyzing a signal that is generated upon the interaction of laser with matter, and thus, it can be considered as a strong candidate for developing an in-situ characterization method. In this work, we propose a method that uses LIBS in a feedback loop system for real time detection and decision making for adjustment of the lasering process on-the-fly. Further, use of LIBS for automated material segmentation, in the 3D image resulting from consecutive lasering and imaging steps, is showcased.

## Introduction

Ultrashort pulsed lasers offer exceptional nanomachining capabilities, thanks to the athermal ablation enabled by the small timescale of their pulse duration [1–7]. However, the laser machining parameters need to be optimized for each machining work, depending on the desired objective and the material composition that is involved. Although, in principle, the parameters can be optimized for a known composition and a given laser system, oftentimes, the sample is composed of several different material types. Examples include printed circuit boards (PCBs) and integrated circuits (ICs), where the traces and the substrate are formed of different materials which interact drastically differently with the laser. As a result, laser parameters need to be adjusted as the laser machining process flow transitions from one material to another. To achieve an efficient overall machining process, it is essential that such adjustment can happen automatically. Otherwise, with complicated geometries present in many samples, the manual monitoring and controlling of the process will be prohibitive from a time and effort perspective. Specifically, the ability to change the machining process parameters based on the material is of paramount importance in automatic end-pointing applications, where the laser machining process must be stopped when a certain material is exposed to the laser. This requires real-time detection of the material composition during the laser machining process.

Various methods exist that can be used for material characterization. In X-ray diffraction (XRD) [8], which is a nondestructive method, the x-rays are directed at the sample and the scattered rays at specific angles from the sample are collected. The intensity of the peaks diffracted from the sample determines the distribution of the atoms. This method has been used in various applications including the pharmaceutical industry [9–11], forensic science[12–14], and the microelectronics industry [15, 16]. An elaborate review of the XRD technique has been provided in [17]. In X-ray photoelectron spectroscopy (XPS), which is a common method for determining the composition of surfaces and interfaces, a solid surface is irradiated with a beam of X-rays and the kinetic energy of electrons emitted from the material is measured. Counting the number of ejected electrons over a range of kinetic energies generates a photoelectron spectrum. The elemental composition and the chemical and electronic state of the atoms are determined from the intensities of the photoelectron peaks. XPS has been employed for surface analysis in various fields such as corrosion, electronics, nanomaterials, biomedicine, and aerospace. In [18], XPS was used to determine the chemical composition and core-shell structure of the iron oxide nanoparticles. In [19], XPS along with depth profiling was used to characterize transistor gate oxide materials and revealed the heterogeneous layer structure of the film by confirming the presence of NO_2_ species at the substrate interface. Arthur et al. [20] employed XPS to study the interaction of a biomaterial with its surrounding tissues inside a human body. In [21], magnesium powders, used to form near-net-shape parts following powder metallurgy, were analyzed using XPS before and after the sintering process to analyze the variation in their surface chemistry. An elaborate review of the XPS technique has been provided in [22]. Further, the combination of scanning electron microscopy (SEM) and energy dispersive X-Ray spectroscopy (EDS) is a common method for material identification. The EDS technique is used to determine the elemental composition of an area of interest which was visually identified and observed using SEM. An X-ray is emitted when the electrons of this area of interest go from an excited state to the ground state. The elemental composition of the area can be determined using the information from these X-rays. The work presented in [23] offers a new image analysis framework to identify phases and quantify the microstructure of cementitious materials from SEM-EDS hypermaps. In [24], various optical light microscopes have been used, in combination with the SEM-EDS, for the identification of stone tool residues. Time-of-flight secondary ion mass spectrometry (TOF-SIMS) is another method for analyzing surface information. In this method, a mass spectrum is generated by determining the masses of positive or negative secondary ions. This mass spectrum along with the secondary ion images are used to determine the composition, distribution, and molecular information of surface constituents [25, 26].

Despite the useful information that the above-mentioned methods can provide about the material properties of the sample, most of them require interruption of the machining process, often with a further requirement for transferring the sample to another instrument and waiting for a characterization process and analysis to be completed before the sample can be transferred back to the lasering apparatus and for machining to continue. As a result, using these methods for sample material characterization in combination with laser processing defeats the purpose of real-time machining.

A characterization technique that can promise to significantly improve this is laser-induced breakdown spectroscopy (LIBS) [27, 28]. LIBS is a form of atomic emission spectroscopy (AES) that rapidly obtains the chemical composition of the area ablated by the laser [29–31]. In LIBS, typically high-power lasers are used to generate a microplasma. The light emission that follows is then collected with an optical fiber and dispersed with a spectrograph. Finally, the detection is performed with a charged-coupled device (CCD) [32]. LIBS can be used for both qualitative and quantitative measurements of materials, which uniquely positions it to replace or produce equivalent results as methods such as XRD and XPS. LIBS has been used in [33] to determine the lead content in drilling fueled soil. The work presented in [34, 35] leverage LIBS for spectral analysis of mint and betel leaves, respectively. Further, spectral diagnosis of health hazardous toxins in face foundation powders using LIBS and inductively coupled plasma-optical emission spectroscopy (ICP-OES) has been reported in [36]. In addition, the laser can be scanned in a predefined path to produce a spatially encoded-LIBS map, in a process that is comparable to SEM/EDS elemental mapping. Importantly, given that LIBS uses the signal that is generated by the interaction of the laser with matter to characterize the material that is being processed, it promises to eliminate the need for transferring the sample out of the laser processing environment as well as the need for interruption of the machining process for performing the material characterization.

Although many unknowns remain in understanding the LIBS signal generation when ultrashort pulsed lasers are used, several aspects of such process have already been explored by the studies reported in the literature. Wessel et al. were able to create fs-LIBS mapping of micro-crack on titanium aluminide (TiAl) using a femtosecond laser system and a scanning microscope [37]. Cravetchi *et al*. studied the standard deviation of fs-LIBS spectra below 2μJ energy per pulse (EPP) and showed that a 1μs delay after the laser shot can yield about twice as much signal to noise ratio (SNR) compared to the no-delay case [38]. Mildner *et al*. was able to achieve 3 times and 12 times enhancement of fs-LIBS spectral intensity using double pules with 800ps duration on Ti and Al respectively [39].

Feasibility of using LIBS for real-time control of ultrafast laser micromachining has been explored in [40]. Their paper concludes that, compared with a strictly passive machining process without any such feedback control, the LIBS-based system provides several advantages including less damage to the substrate layer, reduced machining time, and more-uniform machining. However, for their demonstration application, namely, fabrication of microheater structures on thermal sprayed materials, quantitative measures that compare passive and LIBS-enabled machining are absent. Further, their method for differentiating materials in composite samples is limited to the case that multiple materials are placed in a layer-wise fashion and application of the method for lateral endpointing has not been discussed. Another very important caveat of their method is that it works based on correlating the collected LIBS signal at the location of interest with the signal from previous LIBS collections with the same lasering system on the exact same materials that comprise the sample. This has multiple drawbacks. First, there is significant dependency on the used lasering system and thus the method is not universal. This is especially important since the LIBS signal often consists of characteristic peaks and noise, where the noise part can significantly vary from one system to another, reducing the correlation between two signals, collected by two laser systems, but from the same material. Second, the accuracy of their method is substantially sensitive to the chemical composition of the materials that comprise the sample. Having not conducted prior signal collection on the exact materials that comprise the sample can thus lead to failure in correctly characterizing the sample. This is while, in many applications, prior testing of the comprising material to establish reference signals is impractical. Even when reference signals are available for the comprising material, comparing the collected signal versus all reference signals to calculate the correlations, as proposed by the method, is computationally expensive and thus defeats the purpose of real-time material detection and decision making.

In the presented work, we propose to leverage LIBS in tandem with laser processing to conduct real-time, in-situ material detection for intelligent recipe control, such as automated end-pointing. LIBS signal is analyzed using an efficient signal processing algorithm for fast detection of material peaks. For endpointing, a feedback loop is established, where the signal collected by the LIBS system will be analyzed with minimal delay by the signal processing algorithm, followed by providing instructions to the laser machining control unit on whether the process must be continued or stopped. Further, we explore the feasibility of leveraging the rapid, in-situ material detection system for spatial mapping of the material composition of composite samples. Particularly, the universal coordinates system that correlate the lasering and imaging components and the temporal synchronization between the LIBS collection and the scanning system will be used to register the optical imaging and material characterization spectroscopy information. A main product of such effort is reconstructed 3D images that are automatically segmented without the need for post-imaging manual or semi-automated annotations.

Examples provided herein on automated material detection, end-pointing and composition mapping with the proposed method promise a breakthrough in the automation of nanomaterial processing. Some areas of application include microelectronics failure analysis, as well as segmentation of 3D images that have been produced using a consecutive laser-delayering/imaging approach, without the need for post-lasering image processing.

## Material and methods

### System overview

**Fig 1:**
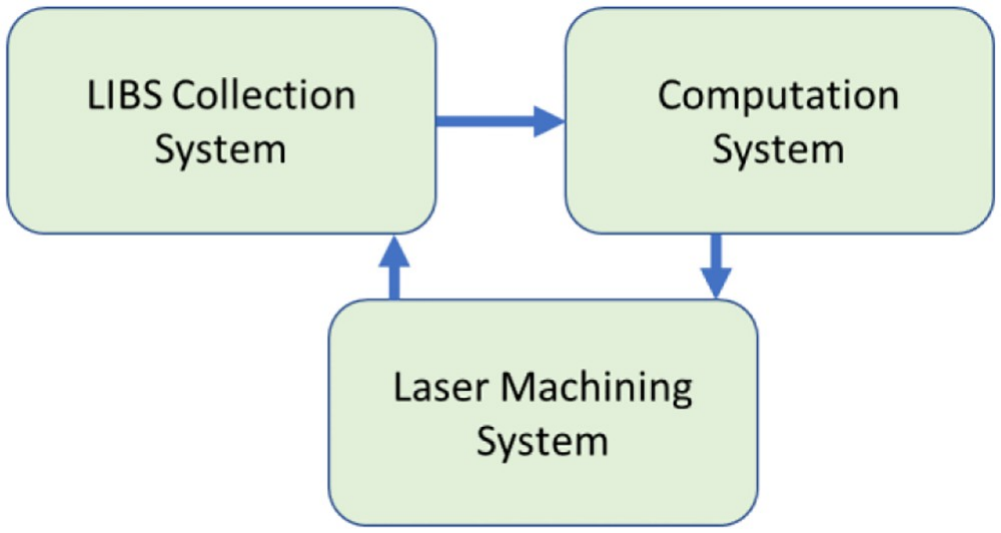
System overview.

When matter is exposed to a laser, a plume is produced, which generates a LIBS signal. This signal is obtained by the LIBS optics and then analyzed by a spectroscope. Subsequently, the analyzed signal is transmitted in real-time to a computer system for swift processing, which allows for timely decision-making regarding the ongoing laser procedure. The computer system constantly provides feedback to the lasering system for uninterrupted process control (Fig 1).

### Laser system

**Fig 2:**
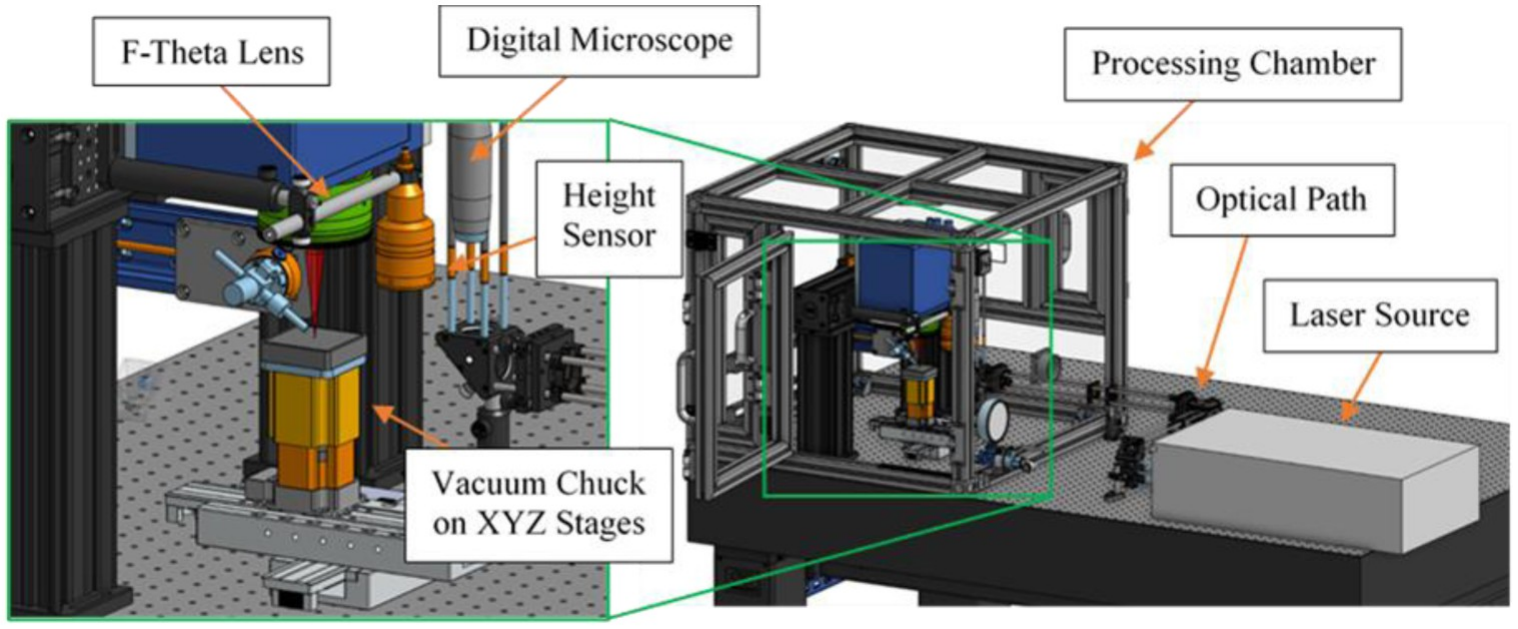
CAD of femtosecond laser machining system.

Coherent Monaco 1035nm 40W laser (1035-40-40) with 257 fs pulse width that can produce a wide range of different pulse repetition rates, from single shots up to 50 MHz, was used in this study. The laser emits a 2.7±0.3mm diameter beam. The beam goes through a beam expander comprised of a fused silica 75mm aspherical lens and a fused silica 300 mm convex lens, which expands the beam diameter to ~11mm. The beam is then delivered to a telecentric fused silica F-Theta lens (TSL-1064-10-56Q-D20) that has an effective focal length of 70 mm. The resulting theoretical spot size with the setup is ~ 8.5 μm. Computer-aided design (CAD) demonstration of the used laser setup is shown in Fig 2.

A typical workflow with the femtosecond machining system starts with precise targeting with a Dinolite digital microscope (AM73915MZT), which is used to identify the region of interest (ROI) first. Zaber LDA series stages (LDA150A-AE53T10A) are used to move the sample precisely on an XY plane. Then the sample is transferred to the Keyence confocal height sensor (CL-P070), which accurately measures the height of the sample up to low micrometer resolution. Zaber VSR series stage (VSR40A-T3A) brings the sample into laser focus. At last, the sample is transferred under the F-theta lens for laser ablation.

### LIBS system

**Fig 3:**
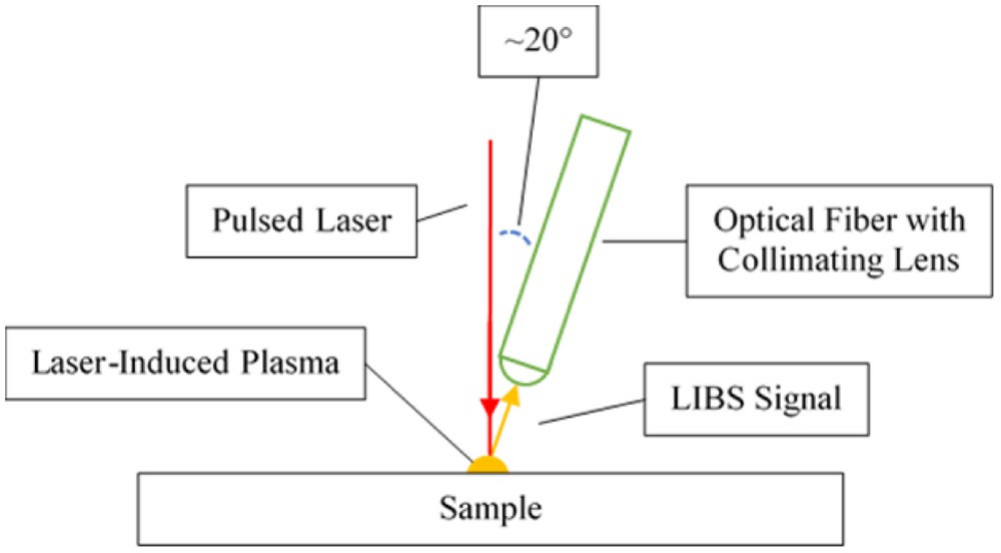
Schematic of LIBS signal collection setup.

Ocean Insight UV-grade optical fiber with a collimating lens (~5mm focal length) is used to collect the LIBS signal. Note that the angle between the laser and the optical fiber is minimized to maximize the efficiency of LIBS signal collection as illustrated in Fig 3. The collected LIBS signal is guided to the Spectral Industries IRIS Echelle spectrometer that disperses the light in two orthogonal directions using two dispersive elements. It is first dispersed through a prism that separates the incoming light into multiple orders and then a diffraction grating that separates the overlapping orders in the orthogonal direction. This creates a ladder of different reflection orders on a complementary metal oxide semiconductor (CMOS) camera that has 1936 x 1216 pixels. The raw experimental result which is shown in Fig 7 is obtained by plotting intensity of CMOS camera pixels against their corresponding wavelengths. The correlation between the camera pixels and the wavelengths are established and verified by using mercury argon lamp (Ocean Optics Hg1). First, the image of the lamp is recorded in such a way that weak peaks are visible while the strong peaks are not saturating the camera. Then the recalibration algorithm from spectral industries is ran based on the obtained image. At last, we verified that the newly found locations of echelle orders on the detector are successfully capturing the spectral peaks. Note that the spectrometer can measure wavelengths in the range of 180–800 & 880-1200nm.

### fs-LIBS measurement

**Fig 4:**
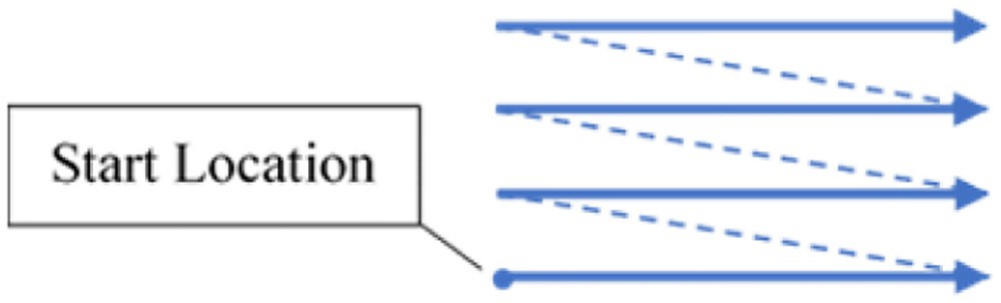
Laser scanning pattern. The arrow indicates the direction of the laser scan, and the dotted line represents the jump line.

To efficiently generate the fs-LIBS signal while minimizing the heat-affected zone (HAZ), a burst of 2 pulses separated by 20ns with a repetition rate of 100kHz is used. Each pulse has a fluence of 20 J/cm^2^. The laser beam is scanned at the speed of 1mm/s in a line pattern, as illustrated in Fig 4. The LIBS system camera is set to a measurement frequency of 20Hz with an exposure time of 50ms.

### ns-LIBS measurement

For comparison, IPG 1064nm 30W nanosecond laser (YLPP-1-150V-30) with 5ns pulse width was also used to generate the LIBS signal. The laser emits a 9±1mm diameter beam which is focused down to about 30μm after going through an f-theta (4401-561-000-26) with an effective focal length of 100mm. To efficiently generate the ns-LIBS signal while minimizing the heat-affected zone (HAZ), laser pulses with a repetition rate of 30kHz and a fluence of 13 J/cm^2^ are used. The laser is scanned at the speed of 1mm/s in a line pattern that is illustrated in Fig 4. The LIBS system camera is set to a measurement frequency of 20Hz with an exposure time of 50ms just like the fs-LIBS scheme. The parameters used for fs and ns LIBS signal generation are listed in Table 1.

**Table 1:** Parameters used for fs & ns LIBS signal generation.

Laser	Spot size (μm)	Repetition rate (kHz)	Fluence (J/cm^2^)	Scanning speed (mm/s)	LIBS Measurement Frequency (Hz)	LIBS Camera Exposure Time (ms)
fs	8.5	100 (with 2 micro bursts, 20ns apart)	20	1	20	50
ns	30	30	13	1	20	50

Aluminum (Al), silicon (Si), titanium (Ti) & copper (Cu), are investigated in this study. Commercially pure materials are used for each element, specifications of which are shown in Table 2. For this work, 53 samples of aluminum with a total of 21053 LIBS datapoints (approximately, 400 LIBS data points in each sample), 59 samples of silicon with a total of 22394 data points, 56 samples of titanium with a total of 22185 LIBS data points, and 59 samples of copper with a total of 21955 LIBS data points were used to optimize the material detection process and to assess the accuracy of detection.

**Table 2:** Specification of materials used for each element.

Element	Material	Composition (%)	Thickness (mm)
**Al**	2024 aluminum	90.75~94.7%	~.508
**Si**	P-type test-grade silicon wafers	N/A	.406~.48
**Ti**	Grade 2 titanium	99.02	~.508
**Cu**	110 copper	99.9	~.508

### Material detection algorithm

**Fig 5:**
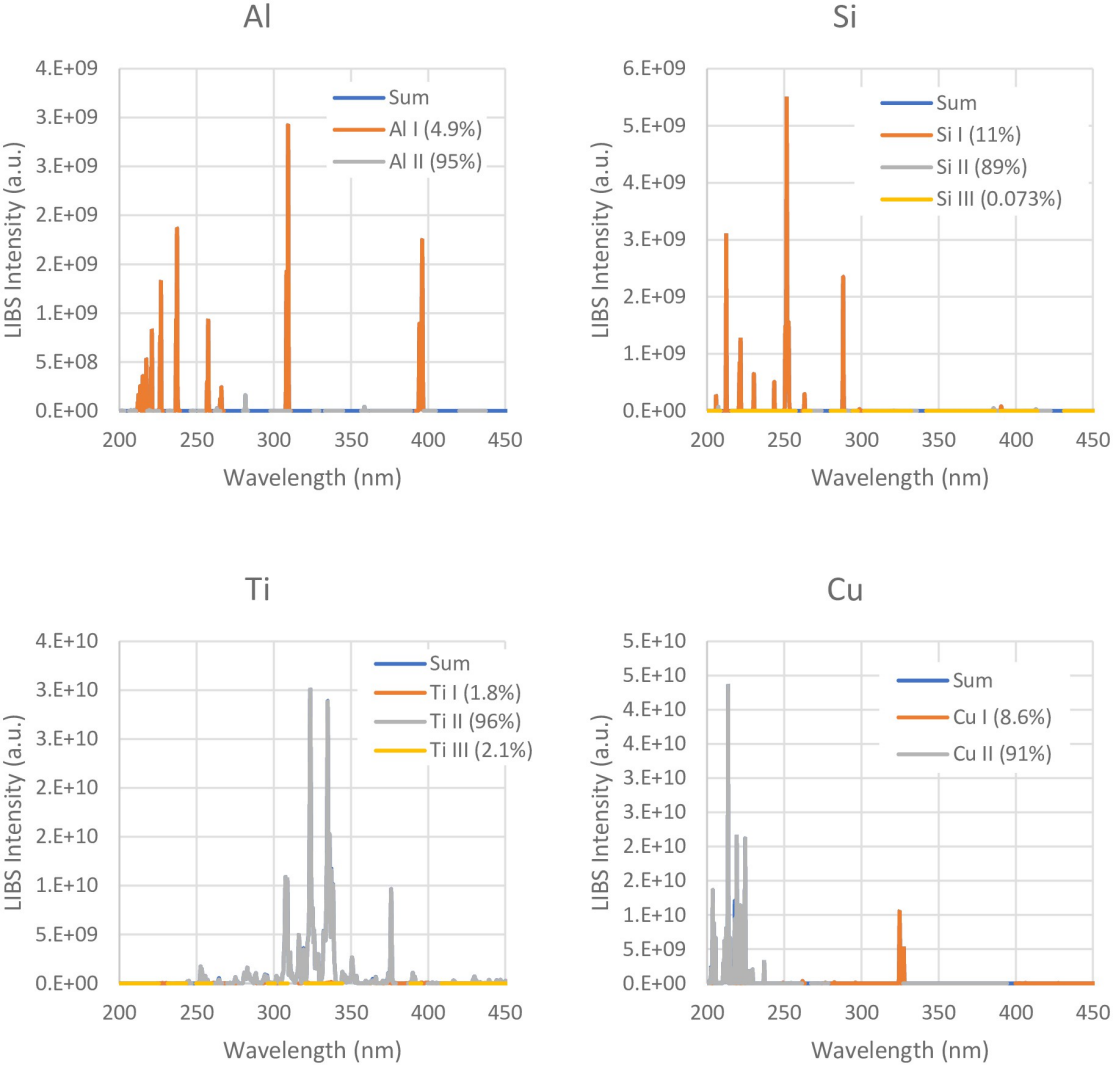
LIBS spectra for Al, Si, Ti, and Cu from the NIST database [41].

The Material detection algorithm relies on the unique spectral peaks exhibited by each material during Laser-Induced Breakdown Spectroscopy (LIBS) generation. These peaks can be identified to accurately detect the material being processed. The NIST LIBS database provides the distinctive spectral peaks for various materials and compositions [41]. Fig 5 shows the expected LIBS spectra for aluminum, silicon, titanium, and copper.

To identify the peaks, our algorithm searches for signals in four different mutually disjoint ranges of wavelengths for these materials. If multiple peaks are detected in different ranges, the one with the highest prominence is selected as the characteristic peak. The prominence of a peak is defined as the relative height of the peak to the lowest contour line encircling it which contains no higher peaks within it.

The algorithm also sets a minimum value for the prominence of the detected peaks. If no peak with higher prominence than this minimum value is detected, it means that either no signal is collected or a signal is produced by a material not listed in Table 2. In this work, the latter situation can be interpreted as the signal being generated while scanning a gap area between the pieces of different materials.

### Endpointing

Laser endpointing can be implemented in various fashions. In all cases, the laser beam scans the sample in a certain pattern, and the generated LIBS spectra are constantly monitored. The lasering will be automatically stopped when a predefined condition in the generated LIBS signal is met. Endpointing conditions can be triggered upon detection of a certain material or otherwise upon lack of it. For example, in a lasering process, Titanium can be the target material, with the endpointing condition being absence of Titanium. In another example scenario, the lasering process can stop whenever the material being lasered is aluminum. The developed algorithm is designed such that, these different scenarios can be easily prescribed by the user, offering a straightforward method for applying automated endpointing.

Currently, the algorithm implemented via python programming language has control over the laser machining system and LIBS spectra collection system. User can easily specify the machining parameters such as energy per pulse (EPP), repetition rate, normal vs. burst mode, scanning speed, pattern and number of repeats via the python user interface. In addition, LIBS measurement parameters such as measurement frequency, exposure time and signal gain are controlled via python code as well. Full list of controllable parameters is shown in Table 3.

**Table 3:** Controllable machining and measurement parameters via python algorithm.

**Machining Parameters**	Laser Parameter	Energy per pulse (EPP)
		Repetition Rate
		Normal vs Burst Mode
	Scanning Parameter	Scanning speed
		Scan Pattern
		Repeats
**LIBS Measurement Parameters**		Measurement Frequency
		Exposure Time
		Signal Gain

**Fig 6:**
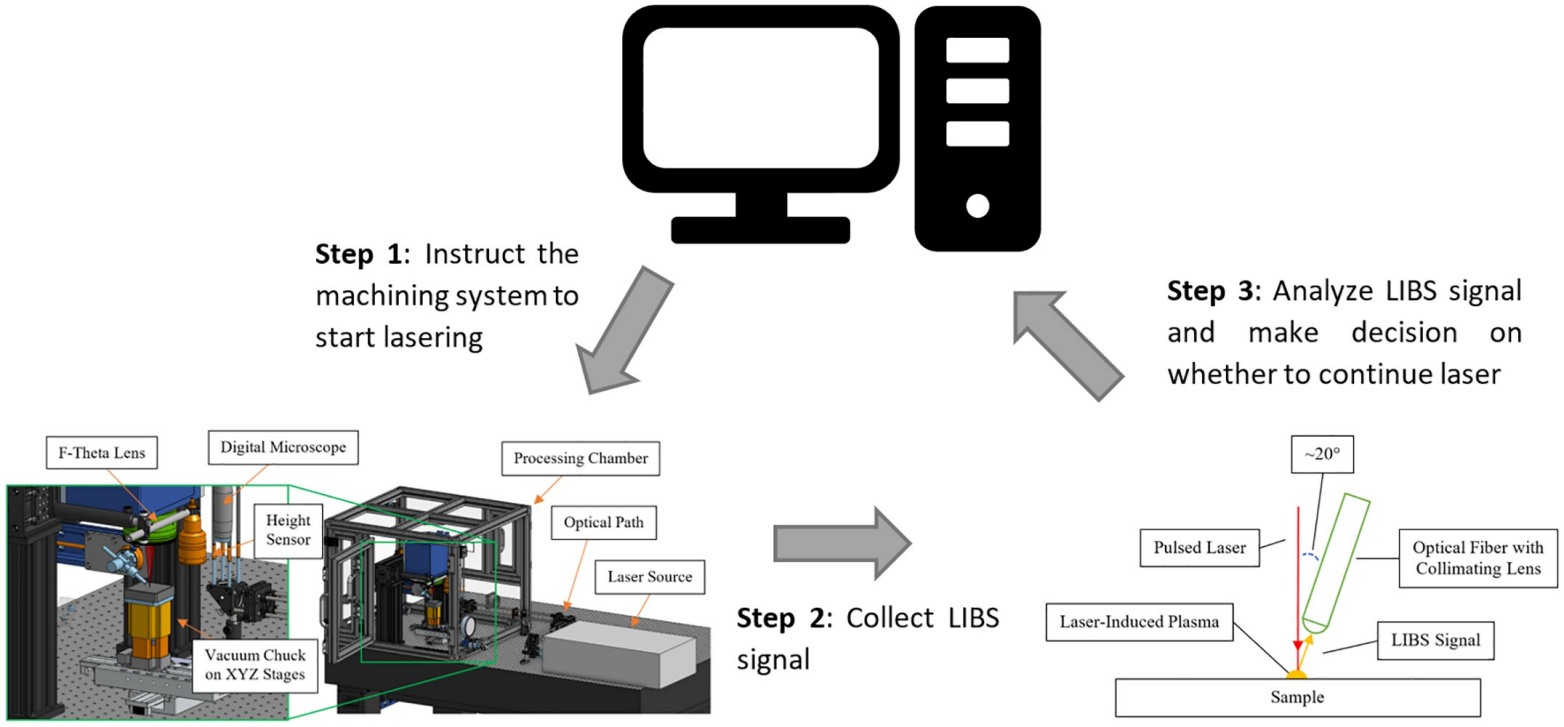
Workflow of endpointing with LIBS. These three steps are repeated over and over again until the stopping condition is met. Note that conditions could enforce either continuation or stopping of the lasering if a certain material is detected.

The endpointing workflow with LIBS is the following: (1) algorithm instructs the laser machining system to start lasering; (2) the LIBS system starts collecting signal and sending the signal back to the computer algorithm; (3) the algorithm analyzes the signal to identify the material being lasered and make decision on whether to continue or stop lasering based on the application; (4) repeat steps 1–3 until the objective is met. (See Fig 6).

Due to the communication and computation time, there is a lag between when the laser hits the material and when instructions are sent about stopping/continuation of the laser machining. The undesirable consequence of this is that there will be a small overshoot of the lasering process on the material, upon detection of which lasering must end. There are two solutions to this problem. First is simply reducing the scanning speed to minimize the amount of such overshoot. But the more elegant solution is the integration of a pulse delay generator (PDG). With PDG, laser can be synchronized with the LIBS collection system and therefore the overshoot can be eliminated.

### Effect of Incident laser energy on LIBS signal intensities

**Fig 7:**
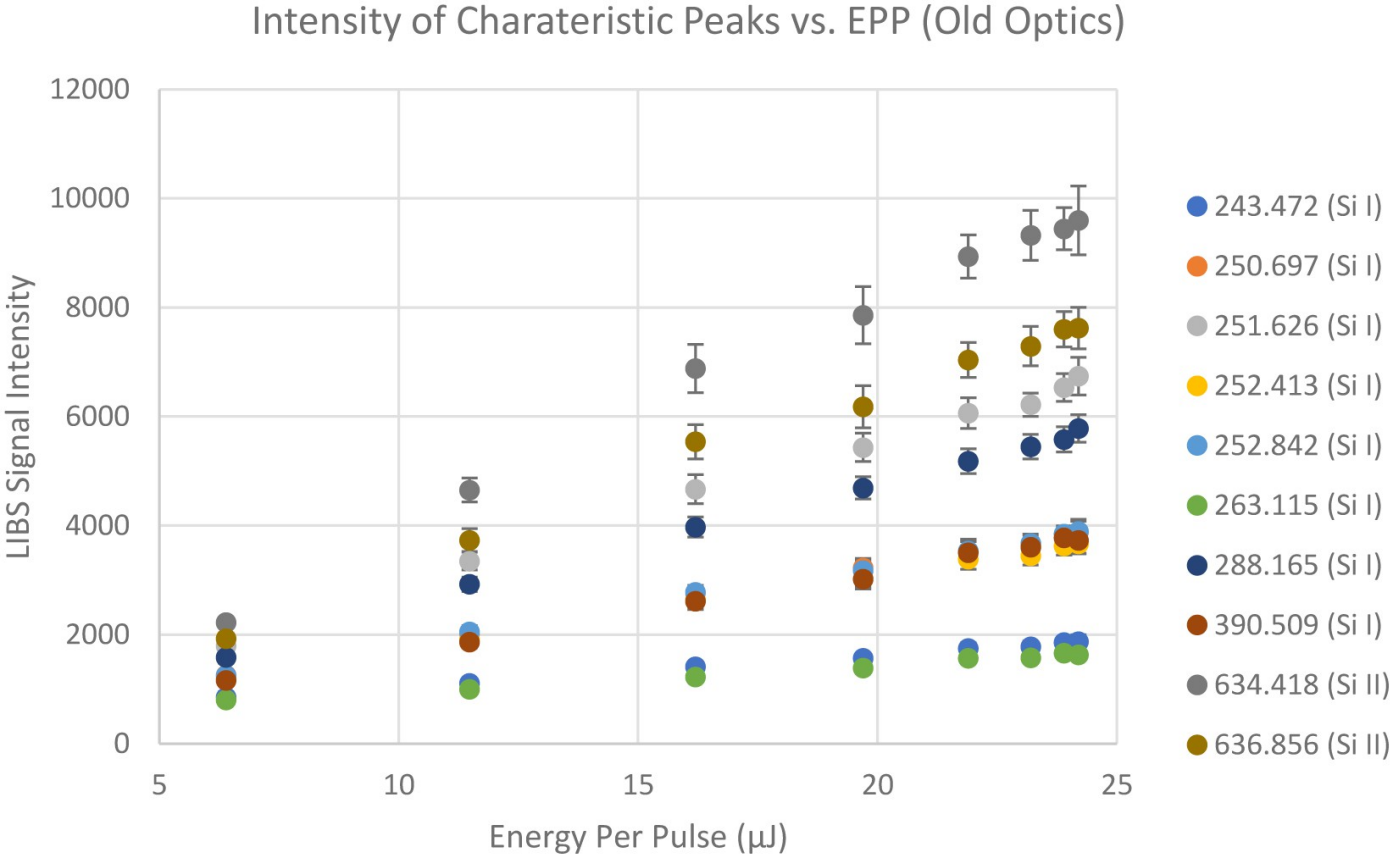
LIBS signal intensities at different wavelengths (in nm) that are close to silicon characteristic peaks from NIST LIBS database collected at normal angle with respect to the sample surface.

To investigate the effect of laser incident energy on LIBS signal intensities, LIBS signals are collected from a silicon wafer at different laser energy per pulse. Then the averages of the LIBS signal intensities near the silicon characteristic peaks collected for a certain period of time are plotted against the laser pulse energy, which is shown in the Fig 7. Notice that all the characteristic peaks’ intensities are increasing linearly as the laser pulse energy increases from 6.39 μJ to 24.2 μJ with strong correlations. All the R-squared values are above 0.9. However, since higher laser pulse energy results in more burrs and heat affected zone (HAZ), the energy per pulse is kept at 6.34 μJ in this study. 6.34 μJ is enough energy to generate sufficient amount of LIBS signal while minimizing the damages.

### Effect of incident laser and collection angle on LIBS signal intensities

**Fig 8:**
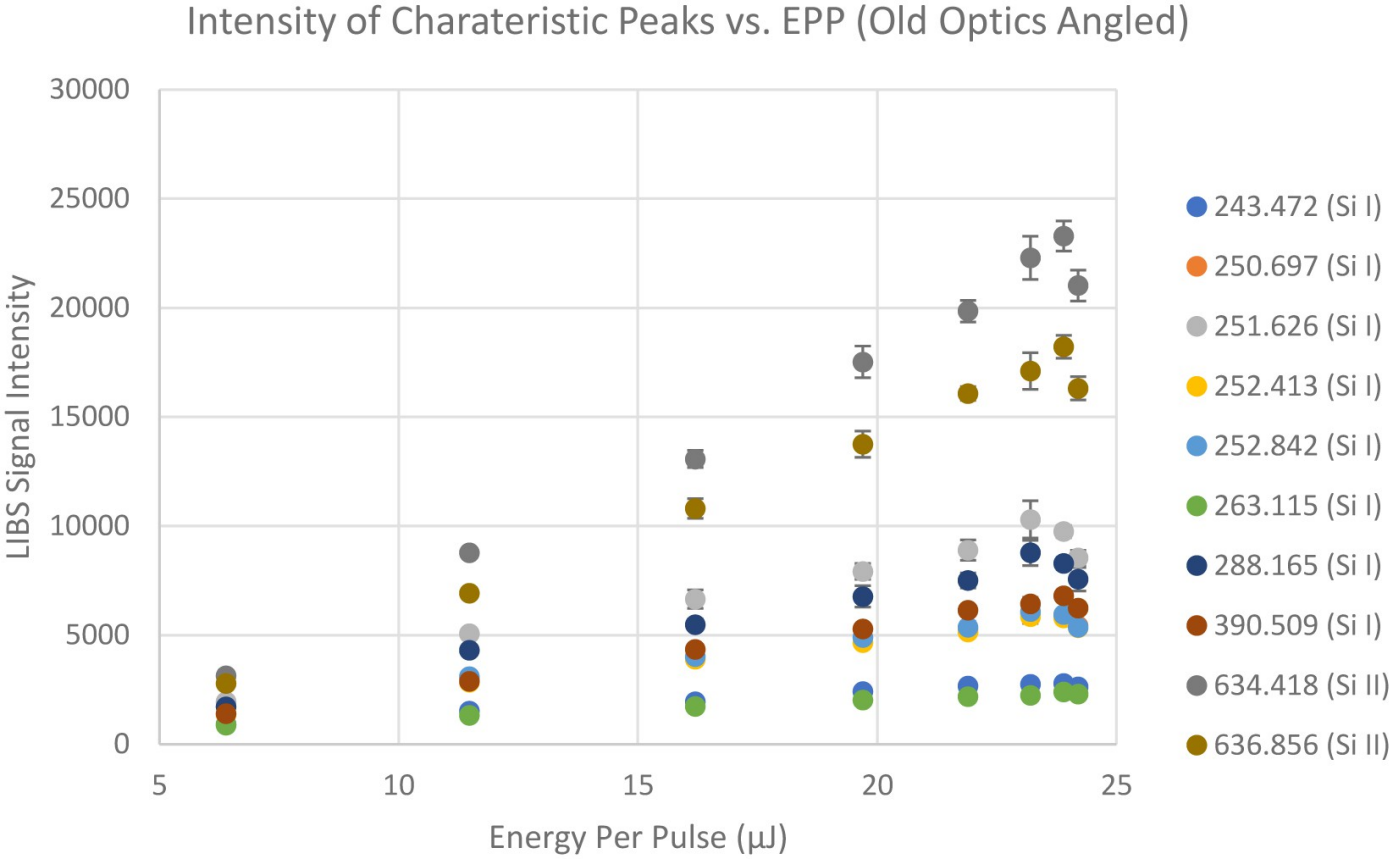
LIBS signal intensities at different wavelengths (in nm) that are close to silicon characteristic peaks from NIST LIBS database collected at 45-degree angle (bottom) with respect to the sample surface.

To investigate the effect of incident laser and collection optics angle, the sample was tilted to a 45-degree angle and laser pulse energy was varied from 6.39 μJ to 24.2 μJ just like the previous experiment. Fig 8 shows the averages of the LIBS signal intensities near the silicon characteristic peaks collected for a certain period of time against the laser energy per pulse. Notice that the overall LIBS signal intensities increased by about 100%.

### Effect of new LIBS signal collection optics

**Fig 9:**
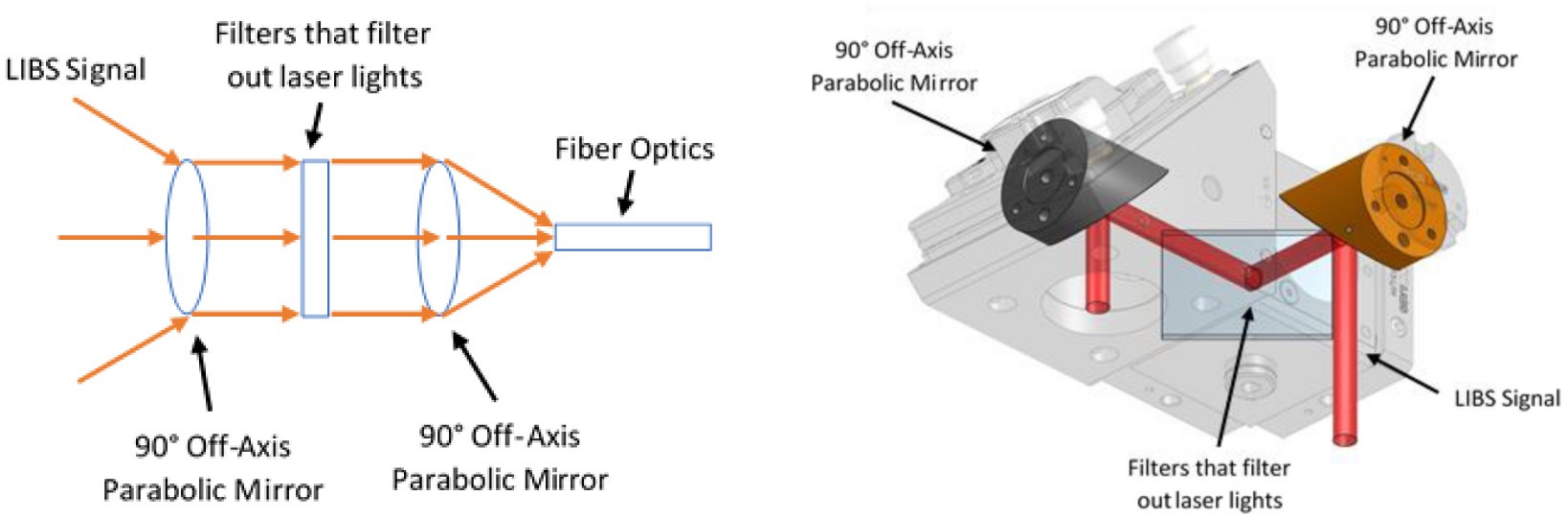
The schematic of different new LIBS signal collection optics is shown on the left and computer-aided design (CAD) of new LIBS signal collection setup is shown on the right.

To increase the amount of LIBS signal collection, larger one-inch optics is used to collimate the scattering LIBS signal, filter out the laser light itself and focused it into the small fiber optics. The computer aided design (CAD) and the schematic of the new optical setup is shown in Fig 9.

**Fig 10:**
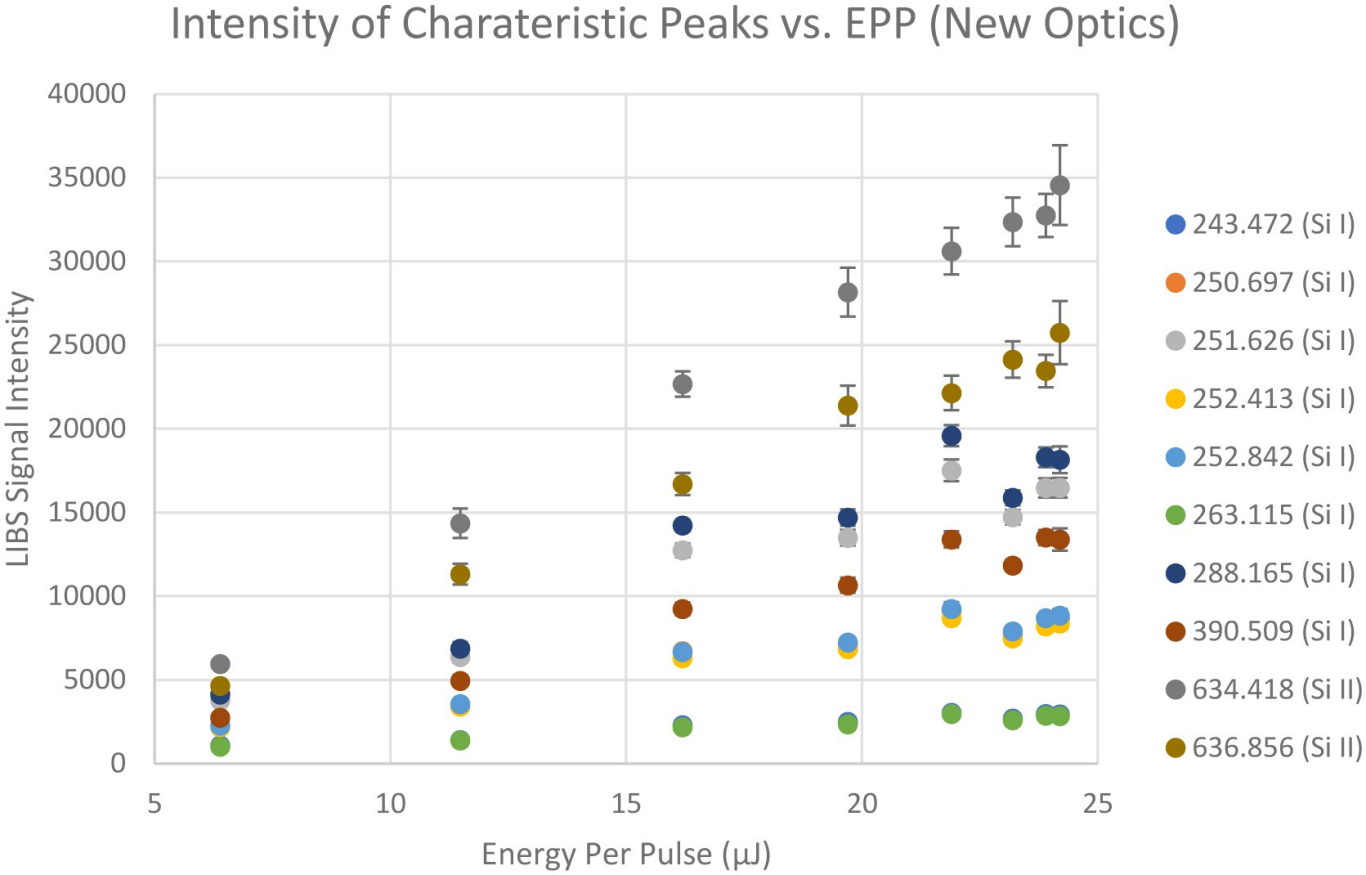
LIBS signal intensities at different wavelengths (in nm) that are close to silicon characteristic peaks from NIST LIBS database collected with newly designed optical setup.

With this improved collection setup, same experiment that was shown previously is repeated and its result is shown in Fig 10 shown below. Notice that the average intensities improved by 250% from the old optical setup.

### Computer model of laser setup and collection optics

**Fig 11:**
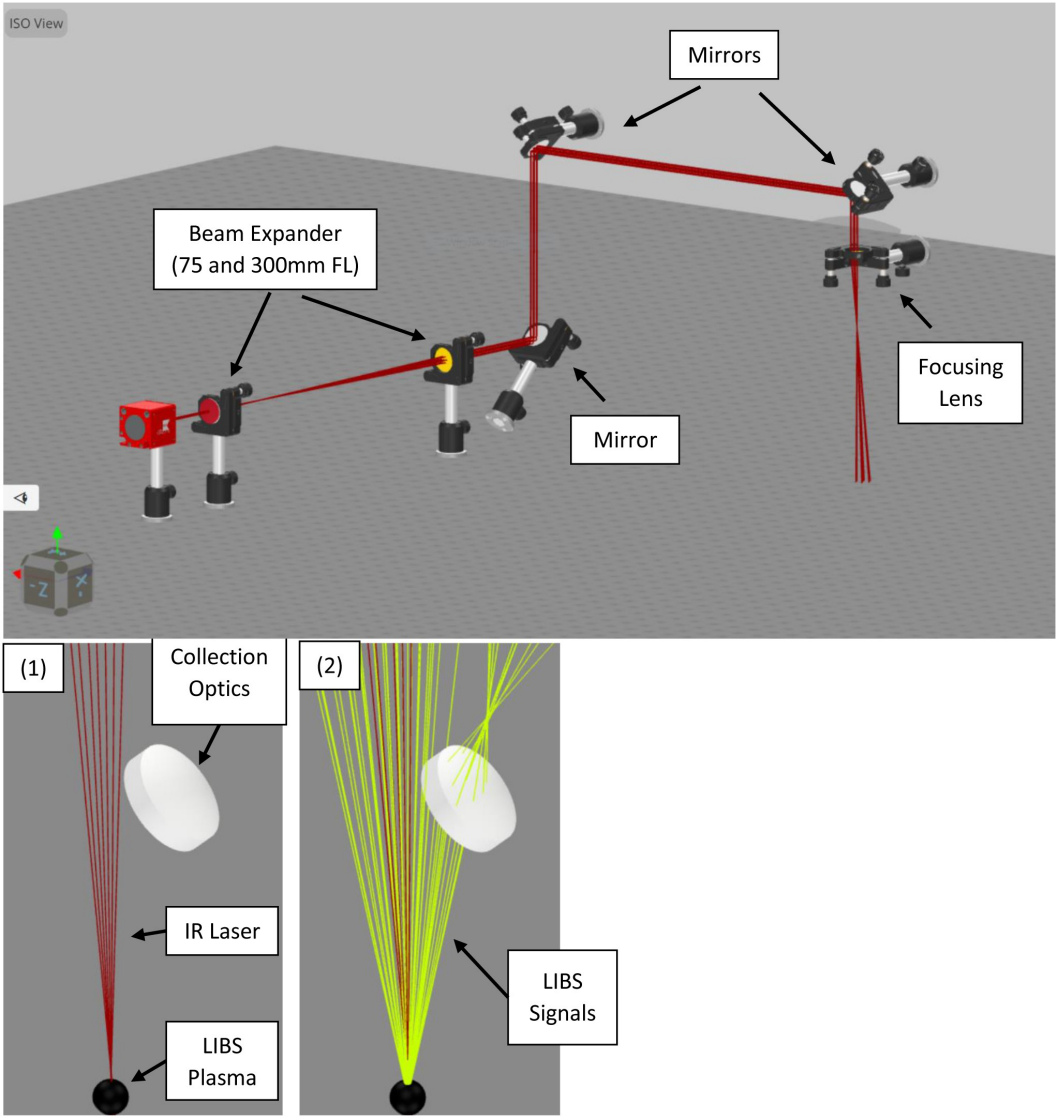
A 3D model of the laser optics and the LIBS signal collection setup in 3DOptix simulation environment.

A 3D model of the laser optics and the LIBS signal collection setup in 3DOptix simulation environment is provided in Fig 11.

#### Comment

In LIBS, to study spectral emission it is recommended to accomplish the optimization of various parameters. Did the author perform any other reference optimization study?

#### Response

Thanks for your comment.

Laser parameter optimization, collection optics and collection angle optimization has been studied. Following three plots as shown above is the experimental result of these optimization and they have been added to the materials and methods section.

## Results and discussion

### Material detection via LIBS signal

The algorithm’s parameters, such as the minimum acceptable prominence and peak search intervals, were optimized using the LIBS datapoints obtained from various samples, described in the Material and Methods section. The optimized parameters were then validated to ensure the accuracy and reliability of the detection algorithm in identifying the peaks related to each material within specific wavelength intervals. As mentioned in the methods section, the algorithm selects the peak with the highest prominence, subject to being greater than a certain threshold. If the prominence is not greater than such threshold, the material being analyzed is considered a gap or void.

**Fig 12:**
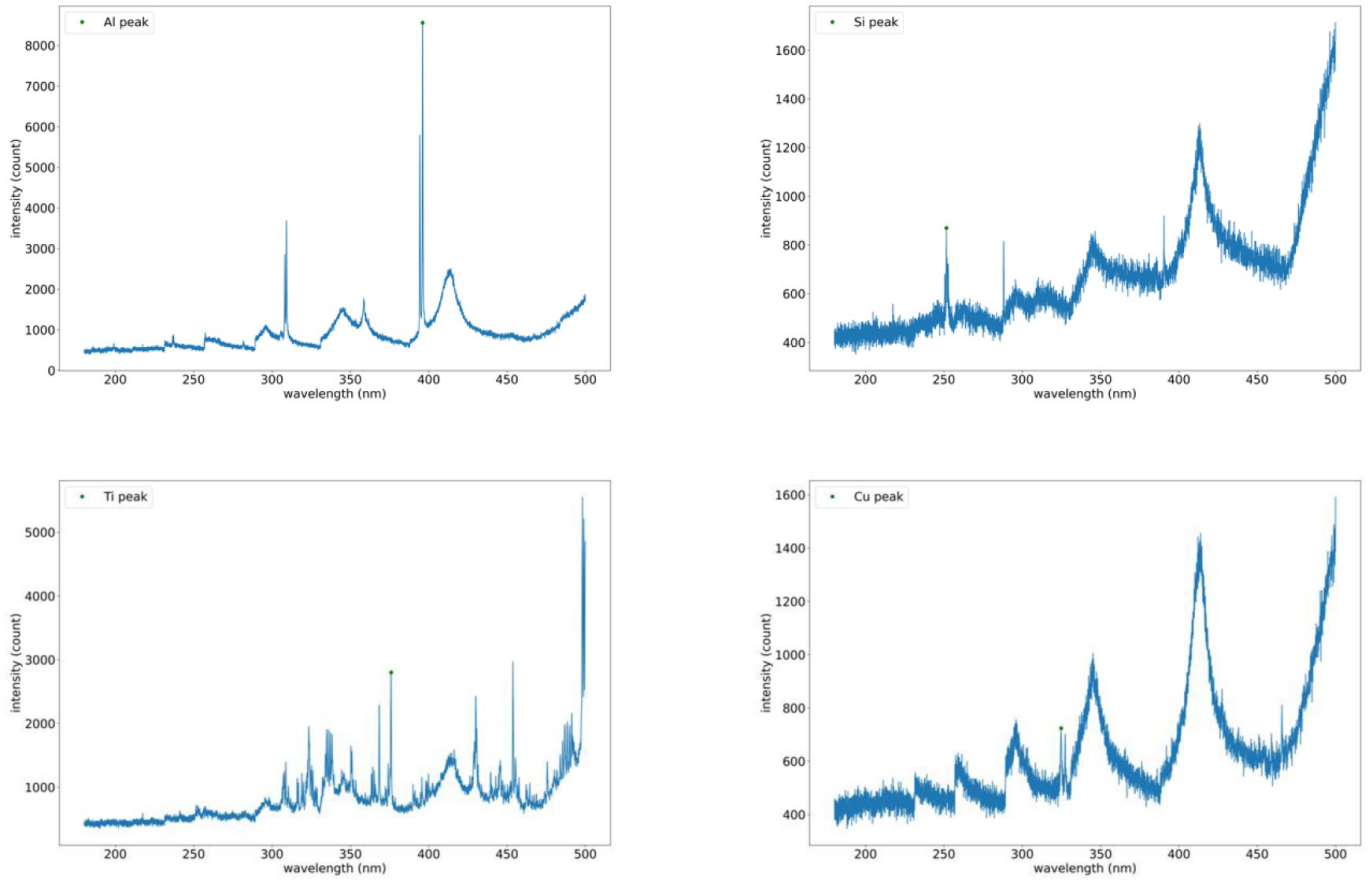
The peaks detected for Al, Si, Ti, and Cu denoted by a green circle.

The algorithm’s searching wavelength intervals for Al, Si, Ti, and Cu were found to be [390 nm, 400 nm], [247 nm, 292 nm], [372 nm, 380 nm], and [320 nm, 331 nm], respectively. The accuracy of the algorithm was determined to be 99.91%, 99.98%, 100%, and 95.91% for Al, Si, Ti, and Cu, respectively. These results demonstrate the effectiveness of the detection algorithm in accurately identifying the peaks associated with each material. Fig 12 displays example spectra acquired for aluminum, silicon, titanium, and copper and shows the detected peaks for each case.

### Automated endpointing

**Fig 13:**
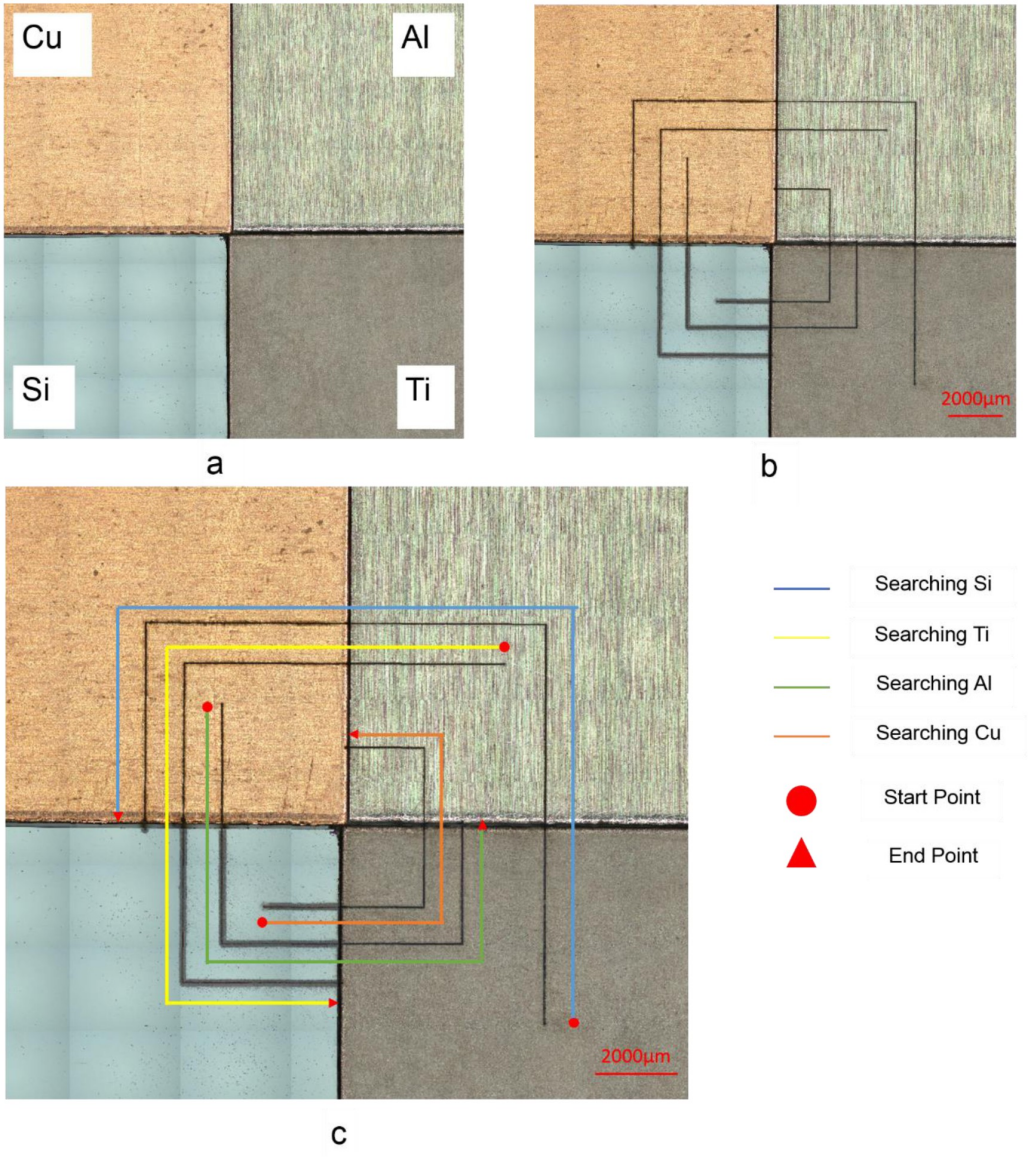
(a) Before & (b) after optical images of a sample with four different materials (Si, Al, Cu & Ti) that were lasered in a rectangular spiral pattern; (c) The laser search lines and the material each line was searching for are Illustrated.

To demonstrate the end-pointing capabilities, a sample with four different materials (Si, Al, Cu & Ti) was created as shown in Fig 13a. The sample was lasered in a rectangular spiral pattern where each laser line was searching for a different material. The first line started from the bottom left corner, in the Al region, and was scanned in a counterclockwise direction until the Si was detected by the LIBS system, per the prescribed endpointing instructions. The second line started from the bottom right corner, in the Cu region and was scanned counterclockwise until Al was detected. The other two searches started from the top right corner and the top left corner, namely the Ti and Si regions respectively, and they were searching for Cu and Ti respectively. This was repeated one more time to demonstrate the repeatability of the end-pointing capability. The automated endpointing was successful and each laser line was able to stop at the desired material region. The resulting laser search lines are shown in Fig 13b, and 13c annotates the pathway of laser lines and the material each laser line was looking for.

**Fig 14:**
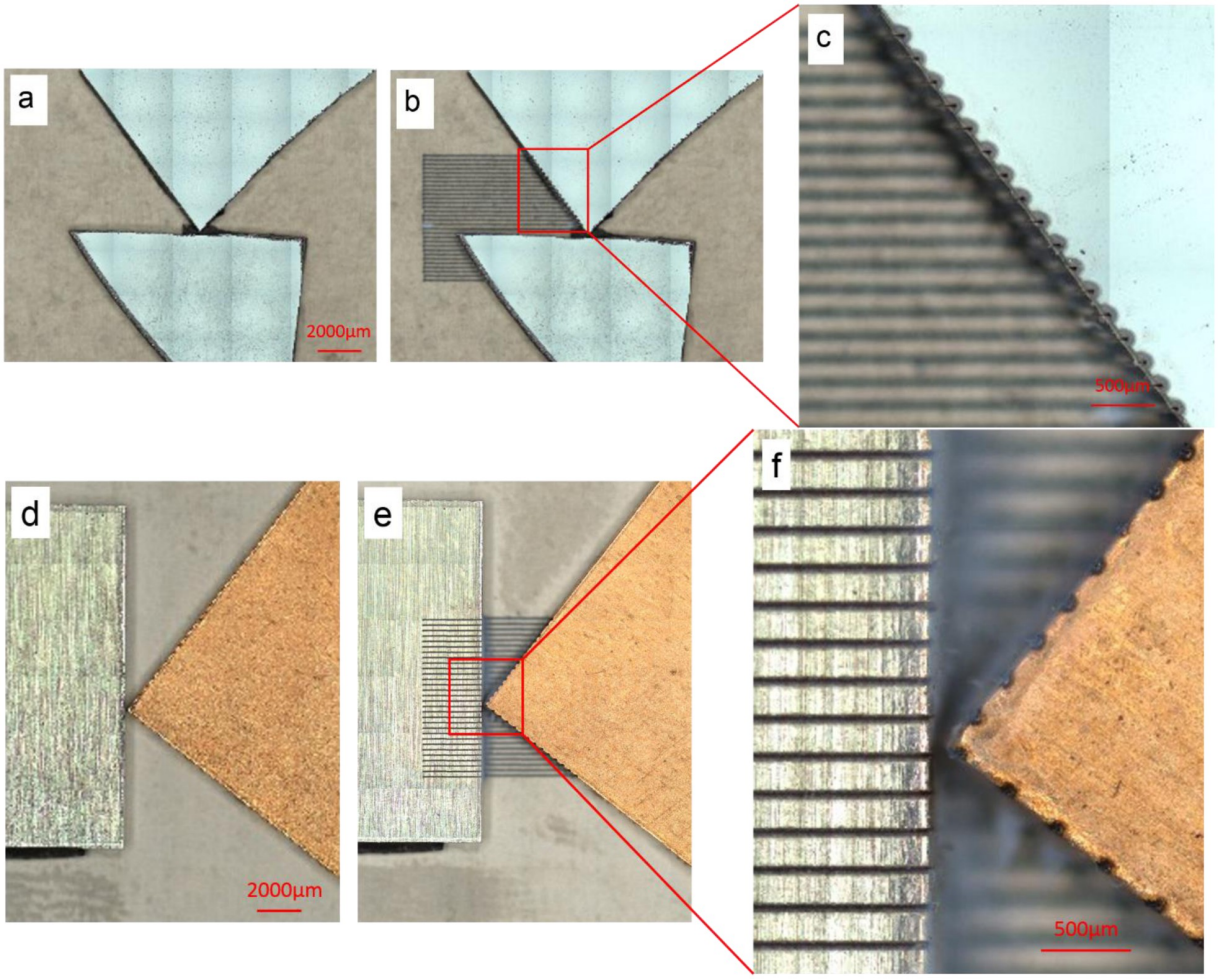
Automated endpointing with the objective of creating parallel laser lines, while remaining in the boundaries of one material region: (a)-(c) before, after, and zoomed-in after lasering images for the stop condition being set as Si; (d)-(f) before, after, and zoomed-in after lasering images for the stop condition being set as Cu.

The endpointing capability was further demonstrated in another set of examples with the objective of creating parallel laser lines, while remaining in the boundaries of one material region. Fig 14 depicts the results for two such examples.

**Fig 15:**
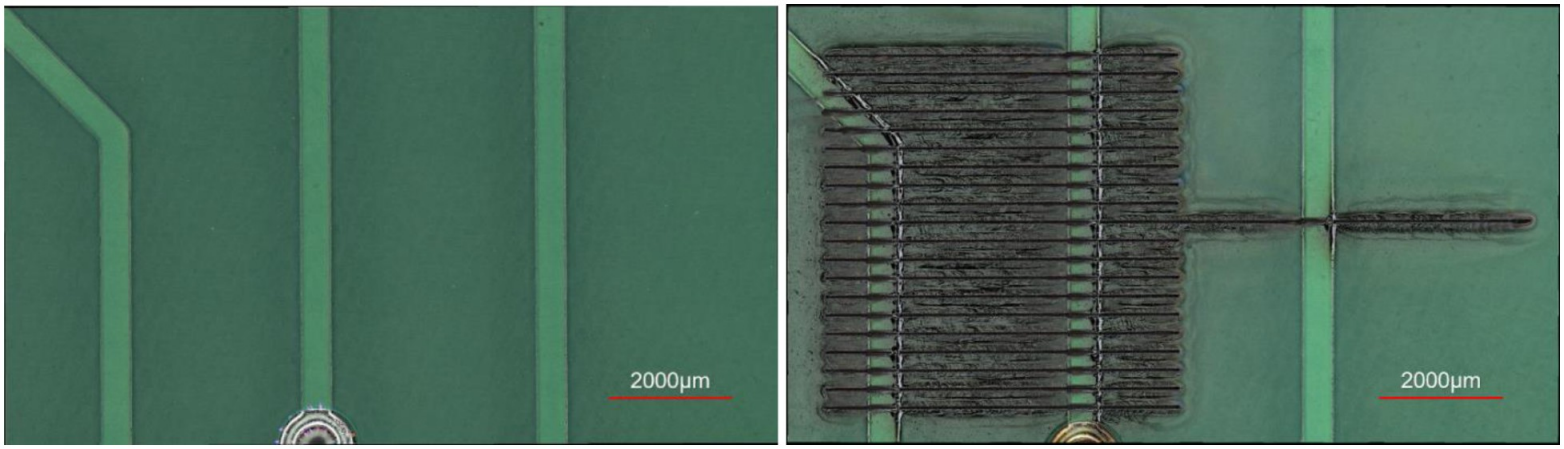
The PCB before and after being lasered.

One added benefit of collecting and monitoring the LIBS signal throughout the laser machining process is that once the temporally recorded LIBS signal is matched with the spatial coordinates of the laser path, the elemental map of the scanned surface can be created. Such capability was showcased on a printed circuit board (PCB) with Cu traces and dielectric composite substrate. A set of parallel laser lines were created in a region on the PCB sample, as shown in Fig 15, and the LIBS signal was collected.

**Fig 16:**
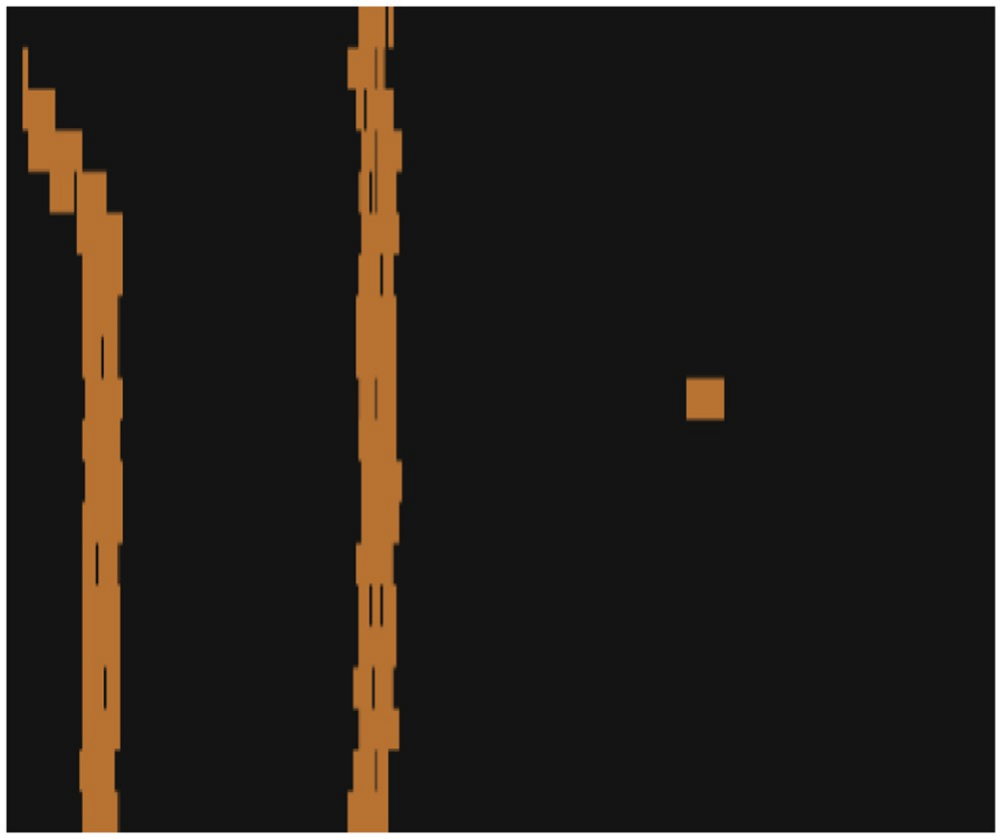
The material composition map for the lasered area on the PCB using the temporally stamped LIBS signals.

Fig 16 displays the spatially mapped LIBS-enabled material detection results for the lasered area. Each pixel is annotated as copper or non-copper. Due to the delay in the generation of the plasma for each material, there is a horizontal shift in the mapped image, which can be corrected for, by applying an offset value. Further, due to the data communication limitations of the current setup, the resulting material map is slightly distorted. This can easily be addressed by employing more efficient data communication hardware. The resulting mapping offers an automated technique for resolving the distribution of materials on composite samples, which promises to eliminate the need for post-process image segmentation algorithms.

## Conclusion

The presented work proposes a novel technique for automated, real-time material detection in ultrashort pulsed laser machining, using laser-induced breakdown spectroscopy. The used setup for creating a LIBS-enabled feedback loop for automated controlling of the laser machining process has been described and the real-time LIBS signal processing has been elaborated. Further, the performance of the proposed technique has been showcased through several examples for end-pointing and material segmentation applications. The findings of this paper suggest that the integration of LIBS into the laser machining platforms, not only can offer substantial improvements in the process automations, by offering endpointing capabilities, but also can significantly reduce the need for post-machining image processing, by providing automated material composition mapping.

## Supporting information

S1 Data (XLSX)
